# Supplementary material for: Multiple ABCB1 transcriptional fusions in drug resistant high-grade serous ovarian and breast cancer
Source: Nat Commun. 2019 Mar 20;10:1295. doi: 10.1038/s41467-019-09312-9 (PMC6426934; doi:10.1038/s41467-019-09312-9)
Supplement: Supplementary file 1 — Supplementary Information [file 41467_2019_9312_MOESM1_ESM.docx]

**MULTIPLE *ABCB1* TRANSCRIPTIONAL FUSIONS IN DRUG RESISTANT HIGH-GRADE SEROUS OVARIAN AND BREAST CANCER**

**Christie et al**

**SUPPLEMENTARY INFORMATION**

This document contains further explanatory details expanding the Results section in order to provide a transparent record of experimental procedures carried out within this study.

Each HGSC recurrent ascites sample was examined for *ABCB1* expression by Q-RT-PCR (primer sequences used in this study are listed in Supplementary Table 1), for 99/108 samples sufficient RNA was available to perform the Q-RT-PCR twice and the expression level was averaged across the two runs. Samples were rank ordered based on their mean expression level of *ABCB1* (Supplementary Figure 1).

**Supplementary Table 1. Primers used in fusion specific RT-PCR and qRT-PCR.**

| **PCR type** | **Target** |  | **Primer (5`-3`)** |
| --- | --- | --- | --- |
| Q-RT-PCR | *ABCB1* | F | GAGAGATCCTCACCAAGCGG |
|  |  | R | CGAGCCTGGTAGTCAATGCT |
| Q-RT-PCR | *GAPDH* | F | AAGGTGAAGGTCGGAGTCAA |
|  |  | R | AATGAAGGGGTCATTGATGG |
| Q-RT-PCR | *ACTB* | F | GCACAGAGCCTCGCCTT |
|  |  | R | GTTGTCGACGACGAGCG |
| Q-RT-PCR | *HPRT1* | F | GTTATGGCGACCCGCAG |
|  |  | R | ACCCTTTCCAAATCCTCAGC |
| Fusion specific PCR 1 | *SLC25A40-ABCB1* | F | CGGCTCTGTGTTGACCAAAC |
|  |  | R | TCTTTGCTCCTCCATTGCGG |
| Fusion specific PCR 2 | *SLC25A40-ABCB1* | F | CCCGTCACCAGGGTTATTCC |
|  |  | R | CCCCTTCAAGATCCATTCCGA |
| Fusion specific PCR | *ARPC1B-ABCB1* | F | CGTCGACTGCCCAGAGTC |
|  |  | R | CCCCTTCAAGATCCATTCCGA |
| Fusion specific PCR | *CLOCK-ABCB1* | F | CACATTGTAAGATGCCTTTGGA |
|  |  | R | CCCCTTCAAGATCCATTCCGA |
| Fusion specific PCR | *PRRC2C-ABCB1* | F | CGAAGTGCGCAAACTTGACA |
|  |  | R | CCCCTTCAAGATCCATTCCGA |
| Fusion specific PCR | *CALU-ABCB1* | F | CTGTGGGGGCTACGAGGAA |
|  |  | R | CCCCTTCAAGATCCATTCCGA |
| Fusion specific PCR | *CNOT4-ABCB1* | F | GAACCCACCGAAAGCTGAGA |
|  |  | R | CCCCTTCAAGATCCATTCCGA |
| Fusion specific PCR | *TMEM243-ABCB1* | F | GGAGGTCATGCTGTCTTGCT |
|  |  | R | CCCCTTCAAGATCCATTCCGA |

**Supplementary Figure 1. Chart of mean *ABCB1* expression level in HGSC relapse ascites samples.**

Each sample was also tested for the presence of the *SLC25A40-ABCB1* fusion transcript using the fusion specific RT-PCR. Twenty-one samples were fusion positive (Supplementary Table 2), the fusion specific RT-PCR was repeated for the 17 samples where sufficient RNA was available. The *SLC25A40-ABCB1* PCR product was confirmed in 15/17 samples. Four of the patients that had a positive result in the first fusion specific RT-PCR had previously undergone WGS and an SV involving *ABCB1* had been described^1^. Based on the combined data, and a requirement for more than one fusion positive result, 17 patients were classified as fusion positive (Supplementary Table 2). The fusion positive patients were spread across the top 64 rank ordered cases (Supplementary Figure 1). Tumours with any fusion partner are marked red.

**Supplementary Table 2. Consistency of *SLC25A40-ABCB1* fusion results.**

We performed WGS on 8 HGSC ascites samples to identify and characterise SVs involving *ABCB1* that may lead to its upregulation. Mean WGS coverage was 39x (range 35-41) for germline and 77x (range 62-85) for tumour samples (Supplementary Table 3). SVs were called by GMS and GRIDSS, except for the 6 cases where WGS was previously performed^1^ for which qSV was run. GRIDSS was run on 2 cases to confirm it called the same *ABCB1* SVs as previously called with qSV^1^.

**Supplementary Table 3. Sequencing metrics.**

| **AOCS Case ID** | **Patient ID** | **WGS mean coverage** | | **RNAseq mapped pairs** |
| --- | --- | --- | --- | --- |
|  |  | **Germline** | **Tumour** | **Tumour** |
| 10335 | 17 | 39.10 | 77.61 | 123,227,827 |
| 10336 | 32 | 40.24 | 77.76 | 122,309,852 |
| 15007 | 91 | 36.70 | 62.14 | 171,004,056 |
| 15257 | 36 | 39.77 | 82.53 | 188,145,352 |
| 15292 | 16 | 34.59 | 83.69 | 176,011,879 |
| 15340 | 7 | 37.00 | 85.26 | 168,331,939 |
| 65547 | 12 | 40.79 | 82.72 | 193,367,802 |
| 15330 | 28 | 40.17 | 61.81 | 192,403,523 |

The SVs called by GRIDSS involving *ABCB1* where the second breakpoint falls within a gene that is not *ABCB1,* those that may cause a transcriptional fusion, are listed in Supplementary Data 2, with all samples bearing SVs involving *ABCB1*. SVs involving *SLC25A40-ABCB1* were identified in 3 patients (Patients 17, 22, & 32), with Patient 17 bearing 2 different SVs leading to a predicted *SLC25A40-ABCB1* fusion. Four novel partner genes for *ABCB1*, where the structure mimics the *SLC25A40-ABCB1* SV with non-coding 5` exons brought adjacent to intron 1 of *ABCB1*, were identified in Patients 7 and 32. The gene partners are: *ARPC1B*, *GTF2I*, *CLOCK* & *STEAP4*. Of these 7 SVs called by GRIDSS, the following were also called by GMS: Patient 7 *ARPC1B*-*ABCB1*, and Patient 17 & Patient 22 *SLC25A40-ABCB1*. The observation that samples had multiple SVs leading to transcriptional fusions involving *ABCB1* indicated that the SVs causing fusion transcripts are subclonal.

In addition to the SVs likely to lead to fusion transcripts, 6 samples (Patients 7, 9, 12, 16, 17, 32) were found to have additional SVs where one breakpoint is within intron 1 of *ABCB1*, however the second breakpoint occurred downstream of the translational start site of the partner gene. These SVs are unlikely to cause functional fusion transcripts. The SV in Patient 9, described previously^1^, involves a 95kb insertion of the 5` end of the *ABCA4* gene (exon 1 – exon 29 out of 50 exons) into intron 1 of *ABCB1*. The 95kb includes ~4kb of non-genic sequence which contains multiple transcription factor binding sites^2,3^ which may contribute to the overexpression of *ABCB1*.

In 7 samples (Patients 12, 16, 17, 28, 32, 36, 91) thirteen SVs had a breakpoint in *ABCB1* that is downstream of the translational start site, again these SVs are not predicted to generate functional fusion transcripts.

Examination of the location of the breakpoints in intron 1 of *ABCB1* found they clustered within a 45kb region at the 3` end of the 113kb intron (Supplementary Figure 2). Interrogation of the sequence of *ABCB1* intron 1 did not identify any known fragile sites (<http://webs.iiitd.edu.in/raghava/humcfs/)> that could explain the frequency of SV breakpoints. We hypothesized that the increased frequency of breakpoints within this 45kb region may be associated with open chromatin, as indicated by the prevalence of DNase I hypersensitivity sites^4^. There are 31 DNase I hypersensitivity sites within the 45kb region harbouring the breakpoints, compared to 56 sites across the entire 113kb of intron 1, and 140 sites across the whole gene (209kb), suggesting that the chromatin of the 45kb region may be more open than the 67kb of intron 1 that does not bear any SV breakpoints.

**
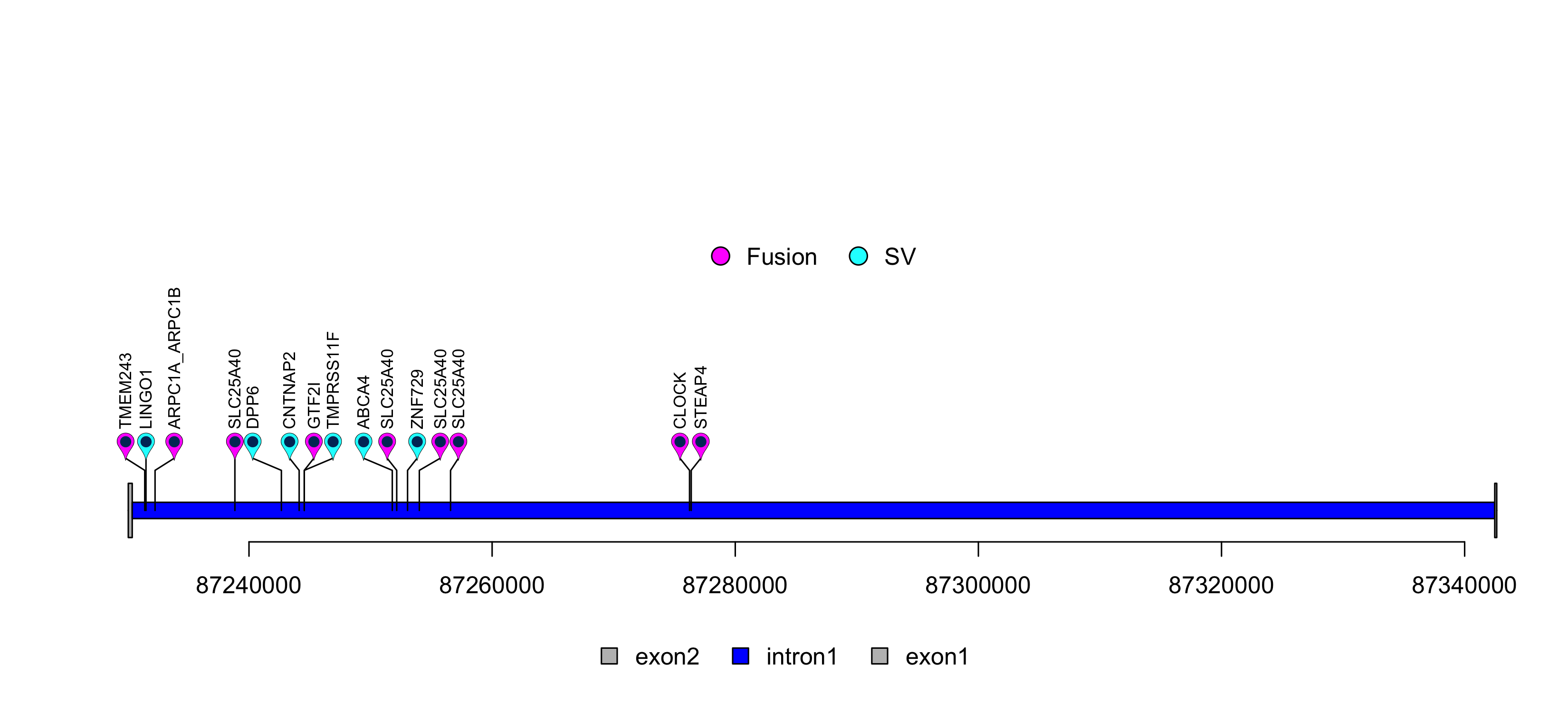
**

Exon 1

**Supplementary Figure 2.** **Location of SV breakpoints in *ABCB1* exon 1.** Breakpoints cluster within a 45kb region in the 3` end of the intron.

Double stranded DNA breaks can be repaired by a number of mechanisms, resulting in different molecular signatures at the breakpoints as discussed by Ottaviani and colleagues^5^. GRIDSS identified homologous sequences at the breakpoints of 7/8 SVs that lead to fusion transcripts, and only in 2/7 non-fusion forming SVs involving *ABCB1* intron 1 (Supplementary Table 4). The homologous sequences at the breakpoints are less than 10bp long, suggesting that the SVs were repaired by microhomology-mediated end joining.

**Supplementary Table 4. Homology at breakpoints identified by GRIDSS.**

We previously examined loci associated with active transposon elements and SVs^1^, including examining commonly active areas in cancer identified by Tubio and colleagues^6^, neither study observed active elements in *ABCB1*.

We performed RNAseq on the same 8 HGSC ascites samples that underwent WGS to characterise fusion transcripts involving *ABCB1*. An average of 166 million paired reads were generated per tumour sample (range 122-193M) (Supplementary Table 3). Fusion transcripts were called with JAFFA and STAR-FUSION, the *SLC25A40-ABCB1* fusion was observed in Patients 17 and 22 but not Patient 32. Additionally, 3 of the 4 fusion transcripts predicted in the WGS SVs were called by at least one caller (Supplementary Table 5). Interestingly, one of the SVs where the breakpoint in the partner gene falls after its translational start site – *TMEM243*-*ABCB1* in Patient 17, was observed to form a fusion transcript in the RNAseq data. This fusion transcript is likely to alter the ABCB1 protein sequence and was therefore not considered further.

**Supplementary Table 5. Consistency of *ABCB1* SV and fusion transcript results.**

We noticed that Patient 9, with the 95kb insertion of *ABCA4* in intron 1 of *ABCB1*, utilised a transcriptional start site in non-coding exon 2 of *ABCB1*. Examination of the RNAseq data from our previous study^1^, found that recurrent HGSC samples more frequently utilised this transcriptional start site than primary HGSC samples which used the transcriptional start site of exon 1, through comparison of the length normalised ratio of exon 2 to exon 1 reads (p < 0.001; Kruskal-Wallis test) (Supplementary Figure 3).


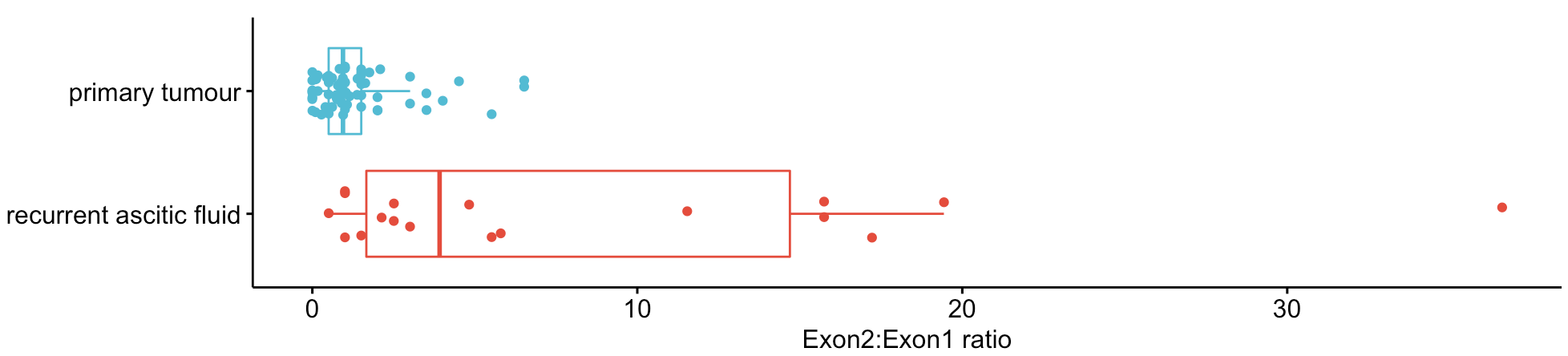


Patient 9

**Supplementary Figure 3. Ratio of *ABCB1* exon 2:exon 1 expression in primary and recurrent HGSC.**

In order to more comprehensively catalogue the range of transcriptional fusion partners to *ABCB1*, we designed an Archer FusionPlex assay to examine 5` partner genes to *ABCB1* specifically (Supplementary Figure 4).

**Supplementary Figure 4. Schematic of the *ABCB1* FusionPlex assay.** Black arrows indicate approximate positions of primers.

The FusionPlex assay was performed on 25 of the recurrent HGSC ascites samples, spread across the top 45 samples ranked by *ABCB1* expression as determined by Q-RT-PCR. A total of 7 samples were found to bear *ABCB1* fusions, including 4 with an *SLC25A40-ABCB1* fusion (Patients 11, 15, 17, 32; Supplementary Data 3). In addition to further validating the *TMEM243-ABCB1*, *CLOCK-ABCB1*, *ARPC1B-ABCB1* and *GTF2I-ABCB1* fusions, 12 novel fusion partners were identified in 4 patients (Supplementary Data 3). Of the 12 novel fusions, only 9 shared the same structure as the *SLC25A40-ABCB1* fusion, whereby non-coding 5` exons were fused to exon 2 of *ABCB1*. The remaining predicted fusion transcripts were not in frame and a premature stop codon would be encountered prior to the *ABCB1* translational start site. Six patients were found to harbor more than one *ABCB1* fusion (Supplementary Figure 5), however aside from the *SLC25A40-ABCB1* fusion only the *CNOT4-ABCB1* fusion was observed in multiple patients.

**Supplementary Figure 5. Number of *ABCB1* fusions identified per patient by FusionPlex.**

To verify some of the non-*SLC25A40-ABCB1* fusions, fusion-specific PCR assays were developed whereby a primer in the partner exon was designed (Supplementary Table 1) and used in combination with the *ABCB1* exon 3 primer previously used. We verified 5 of the novel fusion transcripts by fusion-specific RT-PCR (Supplementary Figure 6).

**Supplementary Figure 6. Fusion-specific RT-PCRs validated novel ABCB1 fusion transcripts.** Patient ID is shown at the top of each gel image. Control (Ctrl) RT-PCRs were targeted to a short region of each transcript not involved in the fusion. NTC – no template control.

While ABC transporters other than *ABCB1* have been linked to chemoresistance, including *ABCC1* (MRP1) and *ABCG2* (BCRP), evidence for their role in human cancer is more limited than that for *ABCB1*. Here we investigated expression, copy number and structural variants in 45 ABC transporter family members (Supplementary Table 6) in primary and recurrent HGSC tumour samples.

**Supplementary Table 6. ABC transporters**

| ABCA1 | ABCA10 | ABCB9 | ABCC9 | ABCF3 |
| --- | --- | --- | --- | --- |
| ABCA2 | ABCA12 | ABCB10 | ABCC10 | ABCG1 |
| ABCA3 | ABCA13 | ABCB11 | ABCC11 | ABCG2 |
| ABCA4 | ABCB1 | ABCC1 | ABCD1 | ABCG4 |
| ABCA5 | ABCB4 | ABCC2 | ABCD3 | ABCG5 |
| ABCA6 | ABCB5 | ABCC3 | ABCD4 | ABCG8 |
| ABCA7 | ABCB6 | ABCC4 | ABCE1 | CFTR |
| ABCA8 | ABCB7 | ABCC5 | ABCF1 | TAP1 |
| ABCA9 | ABCB8 | ABCC6 | ABCF2 | TAP2 |

We accessed WGS, SNP array and RNAseq data that was previously obtained by us and processed to generate expression, copy number and SV calls^1^. As this dataset had complete WGS, SNP, and transcriptome information on all samples, we focused on it rather than adding additional genomic information from the individual samples unique to this study, for which such comprehensive information was generally not available.

The analysis described below made use of a subset of the data from Patch *et al*^1^, focusing on 24 patients with samples collected following the acquisition of drug resistance and two cases with primary ascites samples. Among the 24 patients were 14 cases for which paired sensitive and resistant samples were available (see ^1^ for clinical definitions). Nine of the remaining 10 samples were obtained only at disease recurrence, when the patient was resistant to treatment. One primary tumour sample was included from a chemosensitive patient. A majority of sensitive samples were tumour tissues collected at primary debulking surgery and the resistant samples were mostly ascites-derived tumour cells, collected during disease recurrence (Supplementary Figure 7).

**Supplementary Figure 7. Acquired resistant samples examined for ABC transporters.**

*Primary ascites.

We began by seeking evidence for consistent up-regulation of mRNA expression in resistant disease by performing a t-test for each ABC transporter gene in which we compared sensitive and resistant samples. Four genes, *ABCA1*, *ABCA*7, *ABCA13* and *ABCC2*, were found to have higher expression in resistant samples (Supplementary Figure 8).


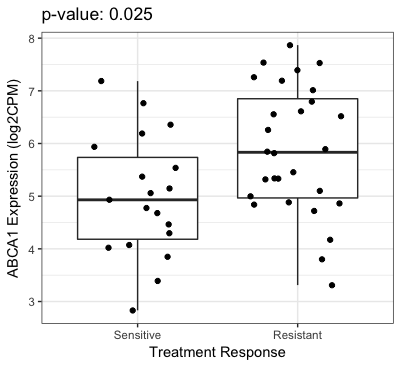

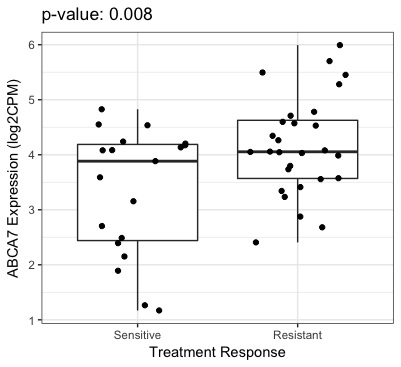

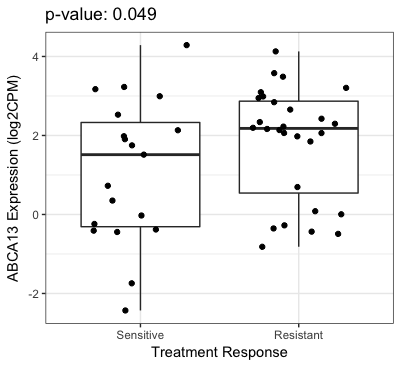

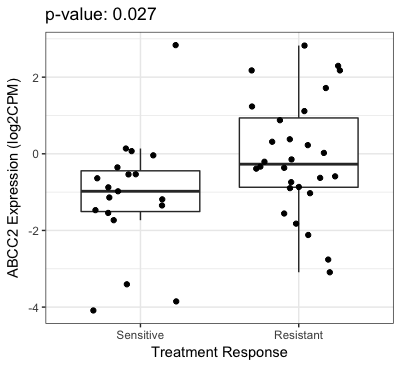


**Supplementary Figure 8. ABC transporter expression in HGSC samples.** t-test; p < 0.05.

As a majority of the resistant samples were ascites and the sensitive samples solid tumours, we considered whether the apparent up-regulation of these genes in resistant samples was a function of sample type. Expression was therefore examined in the 4 cases where primary ascites was available. Due to the limited number of samples no statistical analysis was performed. The expression of all 4 genes showed variability between patients, but no gene showed a pattern of up-regulation in recurrent versus primary ascites samples (AOCS-093 or AOCS-137). The elevated expression of the genes in ascites samples suggests that some of the increased expression in resistant samples could be attributed to the sample type, in particular for *ABCA*7, *ABCA13* and *ABCC2* (Supplementary Figure 9).

**Supplementary Figure 9. Expression of significant genes in 4 cases with primary ascites.** Expression in log_2_CPM, red line at -1 indicates low expression cutoff.

To improve discrimination, we focused on the 14 paired cases and identified 7 genes that were significantly different between sensitive and resistant samples. For all genes, the direction of change was patient dependent; however, for *ABCA12*, *ABCA13* and *ABCC2* expression frequently increased in the resistant sample (Supplementary Figure 10). Examination of AOCS-093 showed that *ABCA13* expression was higher in both ascites samples compared with the sensitive tumour sample, suggesting the apparent increase in its expression in paired samples may be related to sample type rather than resistance *per se*.

**Supplementary Figure 10. Paired analysis.** Expression in log_2_CPM, t-test; p < 0.05. Black arrows indicate AOCS-093 ascites samples, grey arrow indicates AOCS-093 primary tumour sample.

Given the heterogeneity in patterns of expression we used data from *ABCB1* to gain insight the value of expression analysis. It is notable that *ABCB1* expression was not found to be significantly different between sensitive and resistant samples or in an analysis restricted to paired samples (Supplementary Figure 11). However, expression data was annotated for those in which a fusion was present, high ranked expression (Supplementary Figure 11A) or an increase in expression in paired samples (Supplementary Figure 11B) was apparent. We therefore concluded that a combined analysis of expression, copy number and structural variants (SVs), rather than examination of expression patterns alone, was required to identify other transporters of functional significance to acquired resistance.

B

A

**Supplementary Figure 11. Analysis of *ABCB1* in (A) all acquired resistant and (B) paired samples.** Fusion positive samples are indicated by green dots (A) and arrows (B). Expression in log_2_CPM, t-test; p < 0.05.

We examined whether copy number was correlated with expression in the ABC transporter genes. As per our previous analysis of copy number change in HGSC^1,7^, copy number gain was classified when the log_2_ copy number ratio was >0.32 and single copy deletion at a ratio of <-0.32. Fifteen genes showed a significant correlation between expression and copy number; however, copy number gain or loss was only observed for 2 genes: *ABCF2* and *ABCB10* (Supplementary Figure 12).

**Supplementary Figure 12. DNA copy number (log_2_ copy number ratio) vs normalised RNA expression (log_2_CPM).** Pearson correlation, p < 0.05.

Next, we sought tumour samples where an SV breakpoint fell within an ABC transporter, focusing on those SVs that were only observed in resistant samples. Previously, we described 4 cases with SVs in *ABCB1* causing increased expression: AOCS-092, AOCS-150, AOCS-117 and AOCS-120. Additionally, AOCS-135 was observed to bear an SV upstream of *ABCB1* and was *SLC25A0-ABCB1* fusion positive by fusion specific RT-PCR^1^. In addition, structural variants were identified in 9 tumour samples involving *ABCA4*, *ABCA6*, *ABCA7*, *ABCA13*, *ABCB5*, *ABCB7*, *ABCC2*, *ABCC4*, *ABCC9*, and *ABCG2*. We examined the expression of each of the genes in association with presence of an SV. For 6 of the genes, the samples with the SV did not show high levels of expression compared to non-SV bearing samples (Supplementary Figure 13). For *ABCB1*, *ABCA4*, *ABCC2* and *ABCC9* samples with SVs were associated with higher levels of expression (Supplementary Figure 14).

**Supplementary Figure 13. ABC transporter expression and presence of SVs.** Samples with an SV in the gene are shown in green, resistant samples are circles. Expression (log_2_CPM) is rank ordered.

**Supplementary Figure 14. ABC transporter expression and presence of SVs.** Samples with an SV in the gene are shown in green, resistant samples are circles. Expression (log_2_CPM) is rank ordered.

Given the small number of samples with an SV and increased expression, these findings may have occurred by chance and we therefore examined the structure of the SV, seeking evidence of an SV event that may plausibly explain increased expression. The SV in *ABCA4* was observed in AOCS-120/Patient 9, and as described above is a chromosomal translocation where the other breakpoint falls in intron 1 of *ABCB1*. Surprisingly, the SV is associated with high level expression of both *ABCB1* and *ABCA4*, with the RNA read count being very similar before and after the intron 29 breakpoint. *ABCA4* is normally expressed in the retina and to a lesser extent the intestine, kidney and epididymis^8^, and is expressed in some lung adenocarcinomas^9-11^. No chemotherapies are known substrates of ABCA4. Increased *ABCA4* expression is associated with complete response to chemotherapy at diagnosis in HGSC^12^.

The SV in *ABCC2* was observed in one of the metastatic autopsy samples of patient AOCS-167, which also carries a *BRCA2* reversion mutation^1^. The *ABCC2* breakpoint falls in intron 20 (out of a total of 32 exons), involving a chromosomal translocation in which the other breakpoint falls in an intergenic region on chromosome 22, between and downstream of the *SUSD2* and *GGT5* genes, which are in a tail-to-tail direction. Interestingly, the two AOCS-167 samples without SVs show lower *ABCC2* expression than the sample with the SV (Supplementary Figure 15). *ABCC2*, also known as multidrug resistance-associated protein 2 (MRP2), is normally expressed in many tissues including the gastrointestinal tract, liver, endocrine tissues, and with some expression in fallopian tube^8^. It is highly expressed in lung adenocarcinomas^9,10,13^, hepatocellular carcinoma^9,10,14^ and renal clear cell carcinoma^9,10,15^. Chemotherapy substrates of ABCC2 include carboplatin, doxorubicin and paclitaxel, and ABCC2 is known to play a role in chemoresistance^16^.


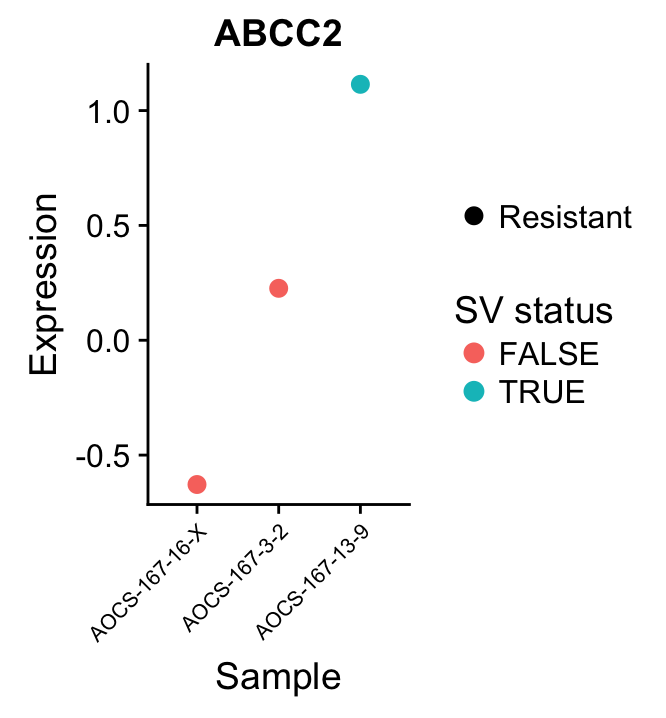


**Supplementary Figure 15. *ABCC2* expression (log2CPM) in AOCS-167 samples.**

The *ABCC9* SVs occur in AOCS-117 and AOCS-142, both unpaired samples. AOCS-117 also has an *ABCB1* SV. The *ABCC9* SV in AOCS-117 is an intrachromosomal rearrangement with the breakpoint falling in intron 14 (out of total of 38 exons) in *ABCC9* and the second breakpoint is in intron 1 of *GYS2*. There is no evidence of a fusion transcript or altered expression around the breakpoint in the RNAseq data. The SV in AOCS-142 is a chromosomal translocation, the *ABCC9* breakpoint is 3.9kb upstream of the transcriptional start site and the second breakpoint is in an intergenic region of chromosome 10 ~15kb upstream of *ARID5B*. *ABCC9* encodes a protein that is a member of the MRP subfamily of ABC transporters that are involved in drug resistance, however ABCC9 itself has no known chemotherapy substrates. *ABCC9* is normally expressed in muscle, liver and female reproductive tissues such as the fallopian tube and ovary^8^, it is also expressed in renal clear cell cancer^9,10,15^, lung adenocarcinoma^9,10,13^, hepatocellular carcinoma^9,10,14^, esophagogastric cancer^9,10,17^ and primary HGSC^9-11^.

It is clear that *ABCB1* is uniquely prominent in structural deregulation associated resistance in HGSC among the family of transporters. Within the limitations of our dataset, chiefly sample size and the potential confounding effect of different tissue types between sensitive and resistant samples, evidence for the involvement of other transporters in drug resistance in HGSC is relatively weak. Using *ABCB1* as a benchmark, it is clear that combining data from SV and expression is most useful. Among the other transporters we did not see recurrent SV that would result in up-regulation of expression of an unaltered protein, as we had for *ABCB1*. The findings with *ABCA4, ABCC2* and *ABCC9* are of most interest, particularly given the previous association of *ABCC2* with drug resistance, however, it is unclear how the SV observed would up-regulate the gene without impacting on protein structure.

As treatment strategies for high-grade serous ovarian cancer partially overlap with those for breast cancer patients, particularly those with *BRCA1/2* germline mutations, we interrogated *ABCB1* in post-treatment samples from 33 breast cancer patients. The cohort comprised 50 samples, collected post-treatment by thoracentesis, biopsy, surgery or during a rapid-autopsy.

As for the high-grade serous ovarian cancer samples, we examined *ABCB1* expression by Q-RT-PCR and looked for the *SLC25A40-ABCB1* fusion transcript by RT-PCR. Due to limiting amounts of RNA, the Q-RT-PCR was only performed twice on 11/50 samples, and the fusion-specific PCR was performed twice on 37/50 samples. As for the HGSC samples, *ABCB1* expression levels varied between patients, therefore samples were rank ordered based on their mean expression level of *ABCB1* (Supplementary Figure 16).


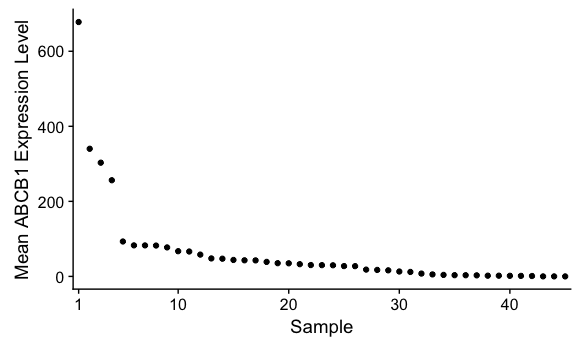


**Supplementary Figure 16. Rank ordered *ABCB1* expression in breast cancer samples.**

Six breast cancer samples were found to positive for the *SLC25A40-ABCB1* fusion in two replicates of the fusion specific PCR, 12 additional breast cancer samples were found to be fusion positive in one PCR replicate (Supplementary Table 7).

The FusionPlex assay was run on 8 breast cancer samples and 3 were *ABCB1* fusion positive (Supplementary Data 4), including one patient that had an *SLC25A40-ABCB1* fusion. The two alternative fusion partners were *NRF1* and *TPX2*. Combining the FusionPlex and RT-PCR results, requiring at least two positive results either from the same or different samples for a patient to be classified as fusion positive, found that 9/33 (27%) breast cancer patients were *ABCB1* fusion positive (Supplementary Table 7).

**Supplementary Table 7. Summary of *ABCB1* fusions in breast cancer patients.** Green dots indicate *SLC25A40-ABCB1* fusion positivity, yellow dots indicate other *ABCB1* fusions, red dots indicate no fusion detected, black indicates the assay was not performed. The final two columns show the summation of the data.

The 6 rapid autopsy breast cancer patients allowed us to examine fusion positivity across multiple metastatic deposits (Supplementary Figure 17). Four of the patients were found to have two out of the three sites tested bearing the *SLC25A40-ABCB1* fusion.

**Supplementary Figure 17. Schematic of locations of *ABCB1* fusion positive cells in breast cancer rapid autopsy patients.** Blue circles indicate fusion positive sites, grey indicates fusion negative sites.

We examined the characteristics of the 15 *ABCB1* fusion partners identified in HGSC and breast cancer patients in an effort to understand why those genes were utilised as partners. We examined each fusion partner for its Gene Ontology (GO) molecular function or cellular compartment, as listed in UniProt ([www.uniprot.org)](http://www.uniprot.org)) (Supplementary Table 8). The GO function for 9 of the partner genes involves binding to nucleic acids, and 5 partner genes bind proteins (Supplementary Table 9). *SLC25A40*, the most common fusion partner to *ABCB1*, performs transmembrane transport.

**Supplementary Table 8. Gene function of fusion partners.**

| **Fusion partner** | **GO molecular function** |
| --- | --- |
| ARPC1B | Structural constituent of cytoskeleton |
| CALU | Calcium ion binding |
| CLOCK | DNA binding transcription factor |
| CNOT4 | Metal ion and RNA binding |
| GTF2I | DNA binding transcription factor |
| ITGB8 | Receptor binding |
| KMT2E | Enzyme and metal ion binding |
| MATR3 | Protein and RNA binding |
| NAP1L1 | RNA binding |
| NRF1 | DNA binding transcription factor |
| PHTF2 | DNA binding |
| PRRC2C | Protein and RNA binding |
| SLC25A40 | Transmembrane transport |
| TPX2 | ATP and protein kinase binding |
| WRN | DNA and ATP binding, helicase activity |

**Supplementary Table 9. Summary of common GO functions for the *ABCB1* fusion partner genes.**

| **Nucleic acid binding** | **Protein binding** |
| --- | --- |
| *CLOCK* | *MATR3* |
| *GTF2I* | *PRRC2C* |
| *MATR3* | *ITGB8* |
| *PRRC2C* | *KMT2E* |
| *CNOT4* | *TPX2* |
| *NAP1L1* |  |
| *PHTF2* |  |
| *WRN* |  |
| *NRF1* |  |

We examined the expression of the *ABCB1* fusion partners as a surrogate measure of their promoter strength. We utilised the primary HGSC and normal fallopian tube RNAseq data from our previous study^1^, in addition to downloading the expression data for the TCGA breast invasive carcinoma study^18^ from cBioPortal ^9,10^, focussing on triple negative samples. For each data set the mean expression level for each gene was calculated and genes rank ordered.

Fallopian tube secretory cells were examined as they are the precursor cell type for HGSC^19^. All of the fusion partners identified in recurrent HGSC were found to have higher expression than *ABCB1* in fallopian tube cells and in primary tumour samples. All of the partner genes observed in breast cancer samples were found to have expression that exceeded *ABCB1*.

We sought to investigate if proximity to *ABCB1* influenced choice of fusion partner. Of the 15 partners observed across HGSC and breast cancer, 9 are located on chromosome 7 with *ABCB1*, the remaining partners are found on other chromosomes (Figure 2b). Of the partners on chromosome 7, they ranged from 66.76Mb 5` to 47Mb downstream of *ABCB1* (measuring from the gene start of each gene as defined by Ensembl). The remaining 6 partner genes were located on chromosomes 1, 4, 5, 8, 12 and 20.

*SLC25A40*, the most common fusion partner, is the closest downstream partner of *ABCB1*. We hypothesize that it would be easiest to generate a fusion of the structure observed by simple deletion for genes that are downstream of and on the same strand as *ABCB1*, rather than complex intrachromosomal rearrangements required for upstream genes or those on the other strand. *RUNDC3B* is closer to *ABCB1* but is not a fusion partner, however *RUNDC3B* is on the opposite strand and has lower expression than *ABCB1* (Supplementary Figure 18).

**Supplementary Figure 18.** (A) Chromosome 7 region surrounding *ABCB1* (from Ensembl). (B) Expression rankings for primary HGSC samples.

We asked whether an increased burden of SVs within a patient was associated with presence of an *ABCB1* fusion. Using the number of SVs per recurrent ascites sample in the Patch *et al* dataset, we found no indication of an association between the frequency of total SVs and the propensity to develop an *ABCB1* fusion (Figure 2c). Additionally, there was no association with the number of intra-chromosomal SVs and presence of a fusion.

A subset of the chemotherapies commonly given to HGSC patients are P-glycoprotein (P-gp, MDR1) substrates (Supplementary Table 10).

**Supplementary Table 10. Commonly used chemotherapies in HGSC.** Data compiled from ^20^.

| **Chemotherapy** | **P-glycoprotein substrate** |
| --- | --- |
| Carboplatin | No |
| Paclitaxel | Yes |
| Liposomomal doxorubicin | Yes |
| Gemcitabine | No |
| Cisplatin | No |
| Bevacizumab | No |
| Cyclophosphamide | No |
| Topotecan | Yes |

All 20 HGSC patients with *ABCB1* fusions had received a P-gp substrate prior to sample collection. Of the 20 fusion positive patients, 18 did not respond to at least 1 line of substrate chemotherapy (Supplementary Figure 19).

**Supplementary Figure 19. Treatment response in patients with an *ABCB1* fusion.** Line 1 treatment often also includes surgery and CA125 response cannot distinguish between effects of the two treatments. Response to substrates are shown in dark green, red and purple, non-substrate chemotherapies are in light green, red and purple. Circle indicates approximate time of sample collection.

We observed a significant association between the number of lines of total chemotherapy and substrate chemotherapy and presence of fusions in HGSC patients (Wilcox test, p-value < 0.001) (Figure 3a). We next investigated whether treatment with certain therapies were more likely to be associated with presence of an *ABCB1* fusion. Significant associations were observed for paclitaxel (p = 0.0048) and carboplatin (p = 0.0435) in HSGC patients (Wilcox test) (Figure 3b, Supplementary Figure 20).

**Supplementary Figure 20. The number of lines of carboplatin treatment in HGSC fusion positive (blue) or negative (red) patients.**

For 3 patients with *ABCB1* fusions we had access to multiple recurrent ascites samples collected during the course of treatment (Figure 3c). We used the *SLC25A40-ABCB1* fusion specific RT-PCR to identify when the fusion was detectable to relate to chemotherapy treatments that selected for cells with the fusion, and examined *ABCB1* expression by Q-RT-PCR.

WGS data from the 23 acquired resistance patients from Patch *et al* was examined to look for co-occurring or mutually exclusive mutations to the *ABCB1* fusions. No significant associations were seen by Fisher’s exact test (Supplementary Table 11), likely due to the small sample size. Analysis of *BRCA1/2* included germline and somatic mutations in *BRCA1/2* and somatic methylation of the *BRCA1* promoter. Forty percent of fusion positive acquired resistant patients had loss of *BRCA1*, compared to 84% of fusion negative patients. *Cyclin E1* amplification, which is associated with platinum resistance, was also considered. Only one acquired resistant patient had a somatic amplification of *CCNE1*, this patient was also fusion positive. Commonly mutated genes in the acquired resistant cohort were also examined for association with *ABCB1* fusion, as per results in Patch *et al*, no significant associations were identified (data not shown).

**Supplementary Table 11. Associations between genomic aberrations and *ABCB1* fusions.**

|  | **Number of Fusion Positive Cases** | **Number of Fusion Negative Cases** | **p-value** | **Log odds ratio** |
| --- | --- | --- | --- | --- |
| ***BRCA1/2* status vs *ABCB1* fusion status** | | | |  |
| *BRCA1/2* mutant/methylated | 2 | 16 | 0.078 | -1.960 |
| *BRCA1/2* wild type | 3 | 3 |  |  |
| ***CCNE1* copy number vs** ***ABCB1* fusion status** | | | |  |
| *CCNE1* amplified | 1 | 0 | 0.208 | Infinity |
| *CCNE1* non-amplified | 4 | 19 |  |  |

For the 108 cases assessed for *ABCB1* in this study, we determined if *BRCA1/2* testing had been performed. Eight of 20 fusion positive patients had germline testing (40%) completed and 4 were mutation positive (50%). Of the 88 fusion negative patients, 32 had undergone *BRCA1/2* testing (36%) and 9 were mutation positive (28%).

We next examined the *in vitro* consequence of *ABCB1* fusions on chemotherapy sensitivity. Previous studies have not observed a correlation between *ABCB1* expression and resistance to taxol, although Parekh and colleagues did observe sensitization to taxol with combination verapamil treatment^21,22^.

A HGSC cell line, AOCS18.5, derived from a recurrent ascites sample that was found to harbor the *SLC25A40-ABCB1* fusion by RT-PCR (Patient 15), was also found to carry the fusion (Figure 4a). This cell line expressed ABCB1 protein as indicated by Western blot (Figure 4b) and immunofluorescence (Figure 4c). An *ABCB1* fusion negative HGSC cell line, AOCS21.2 (derived from the recurrent ascites from Patient 24), did not express ABCB1 protein (Figure 4b). Both cell lines were treated with paclitaxel and cisplatin alone or in combination with a P-glycoprotein inhibitor elacridar. Elacridar treatment resensitised the fusion positive AOCS18.5 cells to paclitaxel but had no impact on sensitivity to cisplatin, and did not impact the IC50 of the fusion negative line AOCS21.2 (Figure 4d, Supplementary Table 12).

**Supplementary Table 12. IC50 values for AOC18.5 & AOCS21.2.**

| **Cell line** |  | **Control IC50** | **Elacridar IC50** | **p-value** |
| --- | --- | --- | --- | --- |
| AOCS18.5 | Paclitaxel | 3.81E-08 | 4.81E-10 | < 0.0001 |
|  | Cisplatin | 1.59E-06 | 1.59E-06 | 0.93 |
| AOCS21.1 | Paclitaxel | 6.08E-10 | 6.53E-10 | 0.77 |
|  | Cisplatin | 5.94E-09 | 5.7E-09 | 0.88 |

# Supplementary References

1. Patch, A.M. *et al.* Whole-genome characterization of chemoresistant ovarian cancer. *Nature* **521**, 489-94 (2015).

2. Rosenbloom, K.R. *et al.* ENCODE data in the UCSC Genome Browser: year 5 update. *Nucleic Acids Res* **41**, D56-63 (2013).

3. Wang, J. *et al.* Factorbook.org: a Wiki-based database for transcription factor-binding data generated by the ENCODE consortium. *Nucleic Acids Res* **41**, D171-6 (2013).

4. Boyle, A.P. *et al.* High-resolution mapping and characterization of open chromatin across the genome. *Cell* **132**, 311-22 (2008).

5. Ottaviani, D., LeCain, M. & Sheer, D. The role of microhomology in genomic structural variation. *Trends Genet* **30**, 85-94 (2014).

6. Tubio, J.M.C. *et al.* Mobile DNA in cancer. Extensive transduction of nonrepetitive DNA mediated by L1 retrotransposition in cancer genomes. *Science* **345**, 1251343 (2014).

7. Etemadmoghadam, D. *et al.* Integrated genome-wide DNA copy number and expression analysis identifies distinct mechanisms of primary chemoresistance in ovarian carcinomas. *Clin Cancer Res* **15**, 1417-27 (2009).

8. Uhlen, M. *et al.* Proteomics. Tissue-based map of the human proteome. *Science* **347**, 1260419 (2015).

9. Cerami, E. *et al.* The cBio cancer genomics portal: an open platform for exploring multidimensional cancer genomics data. *Cancer Discov* **2**, 401-4 (2012).

10. Gao, J. *et al.* Integrative analysis of complex cancer genomics and clinical profiles using the cBioPortal. *Sci Signal* **6**, pl1 (2013).

11. The Cancer Genome Atlas Research Network. Integrated genomic analysis of ovarian cancer. *Nature* **474**, 609-615 (2011).

12. Nymoen, D.A., Holth, A., Hetland Falkenthal, T.E., Trope, C.G. & Davidson, B. CIAPIN1 and ABCA13 are markers of poor survival in metastatic ovarian serous carcinoma. *Mol Cancer* **14**, 44 (2015).

13. Comprehensive molecular profiling of lung adenocarcinoma. *Nature* **511**, 543-50 (2014).

14. Comprehensive and Integrative Genomic Characterization of Hepatocellular Carcinoma. *Cell* **169**, 1327-1341.e23 (2017).

15. Comprehensive molecular characterization of clear cell renal cell carcinoma. *Nature* **499**, 43-9 (2013).

16. Ween, M.P., Armstrong, M.A., Oehler, M.K. & Ricciardelli, C. The role of ABC transporters in ovarian cancer progression and chemoresistance. *Crit Rev Oncol Hematol* **96**, 220-56 (2015).

17. Integrated genomic characterization of oesophageal carcinoma. *Nature* **541**, 169-175 (2017).

18. Cancer Genome Atlas, N. Comprehensive molecular portraits of human breast tumours. *Nature* **490**, 61-70 (2012).

19. Labidi-Galy, S.I. *et al.* High grade serous ovarian carcinomas originate in the fallopian tube. *Nat Commun* **8**, 1093 (2017).

20. Gottesman, M.M., Fojo, T. & Bates, S.E. Multidrug resistance in cancer: role of ATP-dependent transporters. *Nat Rev Cancer* **2**, 48-58 (2002).

21. Parekh, H., Wiesen, K. & Simpkins, H. Acquisition of taxol resistance via P-glycoprotein- and non-P-glycoprotein-mediated mechanisms in human ovarian carcinoma cells. *Biochem Pharmacol* **53**, 461-70 (1997).

22. Gao, B. *et al.* Paclitaxel sensitivity in relation to ABCB1 expression, efflux and single nucleotide polymorphisms in ovarian cancer. *Sci Rep* **4**, 4669 (2014).
