# Supplementary material for: Multiple ABCB1 transcriptional fusions in drug resistant high-grade serous ovarian and breast cancer
Source: Nat Commun. 2019 Mar 20;10:1295. doi: 10.1038/s41467-019-09312-9 (PMC6426934; doi:10.1038/s41467-019-09312-9)
Supplement: Supplementary file 3 — Description of Additional Supplementary Files [file 41467_2019_9312_MOESM3_ESM.pdf]

Description of Additional Supplementary Files

File Name: Supplementary Data 1
Description: HGSC cohort details

File Name: Supplementary Data 2
Description: Structural Variants (SV) identified in WGS of HGSC samples involving ABCB1

File Name: Supplementary Data 3
Description: Fusion transcripts identified by FusionPlex in HGSC samples involving ABCB1

File Name: Supplementary Data 4
Description: Fusion transcripts identified by FusionPlex in breast cancer samples involving ABCB1
