# Supplementary material for: Multiple ABCB1 transcriptional fusions in drug resistant high-grade serous ovarian and breast cancer
Source: Nat Commun. 2019 Mar 20;10:1295. doi: 10.1038/s41467-019-09312-9 (PMC6426934; doi:10.1038/s41467-019-09312-9)
Supplement: Supplementary file 8 — Reporting Summary [file 41467_2019_9312_MOESM8_ESM.pdf]

nature research

Corresponding author(s): David Bowtell

Last updated by author(s): Feb 12, 2019

Reporting Summary

Nature Research wishes to improve the reproducibility of the work that we publish. This form provides structure for consistency and transparency in reporting. For further information on Nature Research policies, see Authors & Referees and the Editorial Policy Checklist.

Statistics

For all statistical analyses, confirm that the following items are present in the figure legend, table legend, main text, or Methods section.

n/a	Confirmed	
☐	☒	The exact sample size (*n*) for each experimental group/condition, given as a discrete number and unit of measurement
☐	☒	A statement on whether measurements were taken from distinct samples or whether the same sample was measured repeatedly
☐	☒	The statistical test(s) used AND whether they are one- or two-sided<br>*Only common tests should be described solely by name; describe more complex techniques in the Methods section.*
☒	☐	A description of all covariates tested
☒	☐	A description of any assumptions or corrections, such as tests of normality and adjustment for multiple comparisons
☐	☒	A full description of the statistical parameters including central tendency (e.g. means) or other basic estimates (e.g. regression coefficient) AND variation (e.g. standard deviation) or associated estimates of uncertainty (e.g. confidence intervals)
☐	☒	For null hypothesis testing, the test statistic (e.g. *F*, *t*, *r*) with confidence intervals, effect sizes, degrees of freedom and *P* value noted<br>*Give P values as exact values whenever suitable.*
☒	☐	For Bayesian analysis, information on the choice of priors and Markov chain Monte Carlo settings
☒	☐	For hierarchical and complex designs, identification of the appropriate level for tests and full reporting of outcomes
☒	☐	Estimates of effect sizes (e.g. Cohen's *d*, Pearson's *r*), indicating how they were calculated

Our web collection on statistics for biologists contains articles on many of the points above.

Software and code

Policy information about availability of computer code

Data collection	No software was used for data collection.
Data analysis	As described in the methods, SNP array data was analysed with published tools GenomeStudio, ASCAT & qPURE. WGS data was analysed with published tools BWA-MEM, GMS and GRIDSS. RNAseq and FusionPlex data was analysed with published tools HISAT2, RNA-seQC, Rsubread, edgeR, Limma, JAFFA and STAR-Fusion. All sequence data was reviewed with IGV.

For manuscripts utilizing custom algorithms or software that are central to the research but not yet described in published literature, software must be made available to editors/reviewers. We strongly encourage code deposition in a community repository (e.g. GitHub). See the Nature Research guidelines for submitting code & software for further information.

Data

Policy information about availability of data

All manuscripts must include a data availability statement. This statement should provide the following information, where applicable:

- Accession codes, unique identifiers, or web links for publicly available datasets
- A list of figures that have associated raw data
- A description of any restrictions on data availability

The whole genome and transcriptome sequencing data will be deposited in the European Genome-phenome Archive (EGA). Most of the data will be publicly available, the germline data will not be publicly available due to restraints imposed by the ethics committee, requests for further data can be made to the EGA Data Access Committee (DAC).
The Patch et al data is available from the EGA repository under the accession code EGAD00001000877. Primary triple negative breast cancer expression data (n = 123) was obtained from cBioPortal ([http://www.cbioportal.org/], Breast Invasive Carcinoma (TCGA, Nature 2012)).

Field-specific reporting

Please select the one below that is the best fit for your research. If you are not sure, read the appropriate sections before making your selection.

☒ Life sciences ☐ Behavioural & social sciences ☐ Ecological, evolutionary & environmental sciences

For a reference copy of the document with all sections, see nature.com/documents/nr-reporting-summary-flat.pdf

Life sciences study design

All studies must disclose on these points even when the disclosure is negative.

Sample size	No sample size calculations were performed, the cohorts were established using all available samples that met the criteria of being recurrent, post-treatment cancer samples.
Data exclusions	No data was excluded from analysis, cases where insufficient sample was available to verify the findings were described but not considered when calculating frequencies of fusions.
Replication	For qPCR, and fusion-specific RT-PCR 2 replicates of each PCR were performed to confirm findings. Where possible novel fusions identified by sequencing studies were verified by orthogonal methods.
Randomization	Randomization is not relevant, as the work aimed to identify molecular features of patient samples.
Blinding	Blinding was not relevant, as the work aimed to identify molecular features of patient samples.

Reporting for specific materials, systems and methods

We require information from authors about some types of materials, experimental systems and methods used in many studies. Here, indicate whether each material, system or method listed is relevant to your study. If you are not sure if a list item applies to your research, read the appropriate section before selecting a response.

Materials & experimental systems

n/a	Involved in the study	
☐	☒	Antibodies
☐	☒	Eukaryotic cell lines
☒	☐	Palaeontology
☒	☐	Animals and other organisms
☐	☒	Human research participants
☒	☐	Clinical data

Methods

n/a	Involved in the study	
☒	☐	ChIP-seq
☒	☐	Flow cytometry
☒	☐	MRI-based neuroimaging

Antibodies

Antibodies used	MDR1 (Abcam, ab170904), GAPDH (Abcam, ab8245), MDR1 antibody (D3H1Q, Cell Signaling Technology)
Validation	ab17094 has been described in 33 publications and is validated for Western blot (WB) for human.<br>ab8245 has been described in >900 publications and is validated for WB for human.<br>D3H1Q has been described in 7 publications and has been validated for human WB and immunoprecipitation.<br>Immunofluorescence with the D3H1Q antibody was previously published by Kim et al (2016) Exp Mol Med 48:e255.

Eukaryotic cell lines

Policy information about cell lines

Cell line source(s)	Cell lines were derived from recurrent ascites samples collected by the Australian Ovarian Cancer Study.
Authentication	The cell lines were authenticated through STR profile matching to the patient's germline sample.
Mycoplasma contamination	Cell lines were regularly tested for mycoplasma and were negative at the time of experiment.
Commonly misidentified lines (See ICLAC register)	None used.

Human research participants

Policy information about studies involving human research participants

Population characteristics	Population characteristics are shown in Table 1. High-grade serous ovarian cancer patients were 24-81 years of age at diagnosis (mean 59.6). Breast cancer patients were diagnosed at age 33-83 (mean kConFab cases 45.2, Utah cases 54.6), 1 male breast cancer patient was included, all remaining patients were female. For both cancers all grades were included.
Recruitment	Patients were recruited to AOCS and kConFab as previously described (Tothill et al 2008 Clin Cancer Res, Thorne et al 2011 J Natl Cancer Inst Monogr) and listed in methods.
Ethics oversight	Peter MacCallum Cancer Centre & University of Utah

Note that full information on the approval of the study protocol must also be provided in the manuscript.
